# Early intensive versus escalation treatment in patients with relapsing–remitting multiple sclerosis in Austria

**DOI:** 10.1007/s00415-024-12256-w

**Published:** 2024-03-02

**Authors:** Michael Guger, Christian Enzinger, Fritz Leutmezer, Franziska Di Pauli, Jörg Kraus, Stefan Kalcher, Erich Kvas, Thomas Berger, Austrian MS Treatment Registry (AMSTR)

**Affiliations:** 1 Department of Neurology, Pyhrn-Eisenwurzen Hospital Steyr, Sierninger Straße 170, 4400 Steyr, Austria; 2 Medical Faculty, Johannes Kepler University Linz, Linz, Austria; 3 Department of Neurology, Medical University of Graz, Graz, Austria; 4 Department of Neurology, Medical University of Vienna, Vienna, Austria; 5 Comprehensive Center for Clinical Neurosciences and Mental Health, Medical University of Vienna, Vienna, Austria; 6 Clinical Department of Neurology, Medical University of Innsbruck, Innsbruck, Austria; 7 Department of Laboratory Medicine, Paracelsus Medical University and Salzburger Landeskliniken, Salzburg, Austria; 8 Department of Neurology, Medical Faculty, Heinrich-Heine-University, Düsseldorf, Germany; 9 Data Management, Hermesoft, Graz, Austria; 10 Statistics, Hermesoft, Graz, Austria

**Keywords:** Multiple sclerosis, Escalation treatment, Early intensive treatment, Comparison

## Abstract

**Objectives:**

To compare the effectiveness of early intensive treatment (EIT) versus escalation treatment (ESC) in a nationwide observational cohort of almost 1000 people with relapsing–remitting multiple sclerosis (RRMS).

**Materials and methods:**

The EIT cohort started with alemtuzumab (AZM), cladribine (CLAD), fingolimod (FTY), natalizumab (NTZ), ocrelizumab (OCR), or ozanimod (OZA); whereas, the ESC cohort was escalated from dimethylfumarate (DMF) or teriflunomide (TERI) to AZM, CLAD, FTY, NTZ, OCR, or OZA within the Austrian MS Treatment Registry. Patients had to stay on therapy for at least 3 months and up to 16 years. The EIT cohort included 743 and the ESC cohort 227 RRMS patients. We used multinomial propensity scores for inverse probability weighting in generalized linear (GLM) and Cox proportional hazards models to correct for the bias of this non-randomized registry study.

**Results:**

Estimated mean annualized relapse rates (ARR) were 0.09 for EIT and 0.4 for ESC patients. The incidence rate ratio (IRR) in the GLM model for relapses showed a decreased relapse probability of 78% for the EIT versus ESC cohort [IRR = 0.22, 95% CI (0.16–0.30), *p* < 0.001]. Analyzing the time to the first relapse by Cox regression, a hazard ratio (HR) of 0.17 [95% CI (0.13–0.22), *p* < 0.001] revealed a decreased risk of 83% for the EIT group. Regarding sustained Expanded Disability Status Scale (EDSS) progression for 12 weeks, a HR of 0.55 [95% CI (0.40–0.76), *p* < 0.001] showed a decreased probability of 45% for the EIT cohort.

**Conclusions:**

ESC treatment after DMF and TERI revealed a higher relapse and EDSS progression probability compared to EIT in Austrian RRMS patients. Therefore, an early intensive treatment should be started in patients with an active or highly active disease course.

## Introduction

In comparison to placebo groups, dimethylfumarate (DMF) reduced the annualized relapse rate (ARR) in patients with multiple sclerosis (MS) by 44-53% [9, 13] and teriflunomide (TERI) by 32-36% [8, 24]. Both have therefore been approved for treatment of RRMS and are used for a mild to moderate disease course [35]. In respective placebo-controlled trials, fingolimod (FTY) reduced the ARR by 48-54% [3, 21] natalizumab (NTZ) by 68% [28] and cladribine CLAD) by 58% [10, 11]. Alemtuzumab (AZM), ocrelizumab (OCR) and ozanimod (OZA) revealed superior efficacy versus moderately effective comparators [4–7, 18].

ESC approach was defined as escalating from platform treatment (DMF and TERI) to AZM, CLAD, FTY, NTZ, OCR or OZA, as these MS drugs are therefore (and by weighing their benefit-risk profile) approved for an active or highly active disease course [35]. In accordance with this definition EIT approach was defined as starting with AZM, CLAD, FTY, NTZ, OCR or OZA in treatment-naive patients with MS.

Most clinicians wish to resort to an evidence-based decision in the case of treatment algorithms concerning early intensive treatment (EIT) and escalation treatment (ESC), in order to balance benefits versus risks.

Studies evaluating early versus late escalation treatment revealed a better outcome for patients, who had been escalated earlier [2, 16, 19, 26, 32]. Furthermore, many studies showed superior effectiveness for patients treated with EIT compared with the ESC approach [17, 20, 27, 29–31, 34].

However, much of this support stems from observational real-world studies that used historic data and lack safety outcomes or randomized control trials [23].

For this reason randomized control trials, such as DELIVER-MS and TREAT-MS, have been initiated to systemically and prospectively compare escalation versus early intensive treatment approaches [1, 23, 25, 33].

Given these considerations, further investigations, in particular real-life data-based studies are needed.

We here, thus, sought to compare the effectiveness of EIT (AZM, CLAD, FTY, NTZ, OCR, or OZA) and ESC approaches after DMF or TERI in a nationwide observational cohort.

## Materials and methods

### Data collection

The Austrian MS Treatment Registry (AMSTR) [14, 15], established in 2006 to maintain quality control and comply with reimbursement regulations of the Austrian sick funds, allows to obtain clinical data, assess indications, clinical profiles of treated patients, and to monitor safety in real life. As the AMSTR was established in 2006, interferon beta and glatiramer acetate treatments are not included. The AMSTR is part of the dense network of approximately 100 MS centers in Austria, which is constituted by MS clinics from neurological departments and dedicated neurological practices that have been assigned this status by the Austrian Society of Neurology based on defined quality criteria. In addition, prescriptions of disease-modifying therapies (DMTs) for MS are exclusively reserved for MS centers. Thus, prescriptions and treatment documentations are evenly distributed across Austria. The AMSTR is compliant with Austrian laws on bioethics and it was also approved by the ethical committee of the Medical University of Vienna (EC number 2096/2013).

The AMSTR documents anonymous baseline data, including the date of clinical onset of MS and disease duration, relapses in the prior 12 months, Expanded Disability Status Scale (EDSS), magnetic resonance imaging (MRI) activity, and previous DMT. Follow-up data [relapses, EDSS, adverse events (AEs), change, or discontinuation of treatment] are required to be documented every 3–6 months. Because of the structure and requirements of the AMSTR, MRI results were only available at baseline before start of treatment, but not during follow-up. Each relapse has to be confirmed by a neurologist at the MS center and documented in the AMSTR. Documentation also requires date of relapse onset, EDSS, and use/dose of i.v. methylprednisolone treatment. Besides the fact that applying the AMSTR is mandatory for reimbursement, external and independent data monitoring to improve data acquisition, input and management in terms of completeness and plausibility of documented data constitute a special quality feature of the AMSTR.

Out of a total of 6472 patients (as by 22nd Dec 2023) documented in the AMSTR since 2006, we identified two cohorts of patients with relapsing–remitting multiple sclerosis (RRMS), who were treatment naïve and had either started with AZM, CLAD, FTY, NTZ, OCR, or OZA (EIT cohort) or had been escalated from DMF or TERI to AZM, CLAD, FTY, NTZ, OCR, or OZA (ESC cohort) within the AMSTR. To be included in the analysis, patients had to stay on each therapy for at least 3 months. The treatment gap within the ESC approach was limited to a maximum of 180 days.

### Outcome measures

We defined the annualized relapse rate (ARR) under treatment during this period as primary outcome measure. Further outcome measures were time to first relapse and sustained disability progression or regression. Sustained disability progression or regression was defined as an increase or decrease from baseline of at least 1.0 point in the EDSS for patients with a baseline EDSS of 0–5.0 (or at least 0.5 points for patients with a baseline EDSS score greater than 5.5) that persisted for at least 12 or 24 weeks and did not change until the last available EDSS score during the observation period. Therefore, four EDSS measurements were at least required. The EDSS assessments are on average performed every 2–4 months depending on the different monitoring requirements for treatments in Austria.

### Statistical methods

All effects estimated in comparing treatment groups were average treatment effects (ATE). To control the bias for non-randomised assignment to the treatment groups, we used normalized inverse probability weighting (IPW). Propensity scores for treatment in the EIT and ESC cohorts were estimated with a logistic regression model with the baseline parameters age, duration of disease, number of relapses 12 months prior to baseline, EDSS, presence of at least 9 cerebral MRI T2 lesions and at least one contrast-enhancing brain MRI lesion, and previous therapy as independent variables. These variables were included in the model because of their clinical meaning, independent from their significance as a predictor in the model. Therefore, we tried to overcome the problems of being misled by false positive predictors in a multiple testing situation as well as of missing relevant variables by abandoning them in a beta failure decision. The study cohort was truncated for one patient producing weights higher than ten. Treatment groups were well balanced for all variables after weighting.

A generalized linear model (GLM) with relapse count as Poisson distributed dependent variable and logtransformed observation time in years as offset variable was used to estimate the treatment effect on the ARR in the observation period.

Potential means for these changes were estimated for each treatment from the model.

We used Cox proportional hazards models for analysing EDSS progression and regression confirmed after 12 and 24 weeks, and the relapse hazard in the observation period.

All models included treatment as categorical factor and normalized inverse propensity scores as weights regarding the survey character of the study. All variables used for propensity scoring were also used in the outcome models as independent variables to obtain adjusted treatment effects. We applied this double robust approach, because the ATE estimator remains consistent if at least one of the two, the propensity score model or the outcome regression, is specified properly. Thus, the misspecification of only one of the two models would not cause any harm to the ATE estimator [12].

The proportional hazards assumption for the Cox models had been verified by non-significant deviations from the proportional hazards assumption for each covariate in the model using Schoenfeld residuals.

As statistical programs, we used IBM SPSS Statistics for Windows, Version 24.0 (Armonk, NY: IBM Corp.), Stata Statistical Software, Release 17 (College Station, TX: StataCorp LLC.).

## Results

According to the pre-defined inclusion criteria, the cohort of EIT included 743 (AZM: 15, CLAD: 45, FTY: 183, NAT: 283, OCR: 151, OZA: 66 patients) and the cohort of ESC 227 (DMF: 68, TERI: 159 patients) RRMS patients, respectively (Table 1). After escalating from DMF and TERI, 3 patients were treated with AZM, 39 with CLAD, 89 with FTY, 52 with NTZ, 17 wit OCR and 27 with OZA, respectively (Table 1). The EIT cohort corresponds to highly effective treatment approach as cited in the literature [1, 23].

**Table 1:** Treatment distribution of EIT and ESC cohort

Treatment	EIT *N* = 743	ESC before switch *N* = 227	ESC after switch *N* = 227
DMF	0	159	0
TERI	0	68	0
AZM	15	0	3
CLAD	45	0	39
FTY	183	0	89
NAT	283	0	52
OCR	151	0	17
OZA	66	0	27

*AZM* alemtuzumab, *CLAD* cladribine, *DMF* dimethylfumarate, *EIT* early intensive treatment, *ESC* escalation, *FTY* fingolimod, *NTZ* natalizumab, *OCR* ocrelizumab, *OZA* ozanimod, *TERI* teriflunomide

The baseline data of both groups before treatment switch are summarized in Table 2 and showed slight differences for some baseline variables. Normalized IPW resulted in a weighted sample size of 491 and 464 patients, respectively, and this was well balanced indicated by standardized mean differences (SMD) below 0.1, with the exception of the EDSS (SMD = 0.16) (Table 2).

**Table 2:** Patient characteristics at baseline

	EIT *N* = 743	ESC *N* = 227	EIT weighted *N* = 491	ESC weighted *N* = 464	SMD weighted
Female					
* N*	473	152	315	306	− 0.036
%	63.7%	67%	64.2%	65.9%	
Age					
Mean	36.4	34.7	36.1	35.9	0.024
SD	11.3	10.3	11.3	10.2	
Duration of MS (years)					
Mean	4.7	5.4	5.0	4.6	0.058
SD	7.0	6.9	7.5	6.3	
EDSS					
Mean	2.5	1.5	2.3	2.0	0.16
SD	1.7	1.1	1.7	1.3	
Relapse rate within 12 months prior treatment switch					
Mean	1.5	1.1	1.4	1.3	0.062
SD	1.1	0.8	1.0	0.8	
≥ 9 T2 lesions					
*N*	648	199	429	409	0.028
%	87.2%	87.7%	87.3%	88.3%	
≥ 1 Gd-enhancing T1 lesion					
*N*	494	116	308	298	0.031
%	66.5%	51.1%	62.7%	64.2%	

*ARR* annualized relapse rate, *EDSS* Expanded Disability Status Scale, *EIT* early intensive treatment, *ESC* escalation, *Gd* gadolinium, *MS* multiple sclerosis, *SD* standard deviation, *SMD* standardized mean difference

Mean follow-up was 3.3 years (SD 3.0) for unbalanced EIT and 4.7 years (SD 2.1) for unbalanced ESC patients, after IPW 3.3 (SD 3.0) and 4.6 (SD 2.1), respectively.

For the ESC cohort, the mean time to escalate from DMF and TERI was 2.1 years (SD 1.6).

The reasons for escalating therapy within ESC group were mainly inflammatory disease activity (clinical and/or radiological activity; *n* = 305), patients’ wishes (patient’s decision) (*n* = 121) and adverse events (AEs) (*n* = 49). It is important to note that treating neurologists were allowed to name several reasons per patient.

**Fig. 1:**
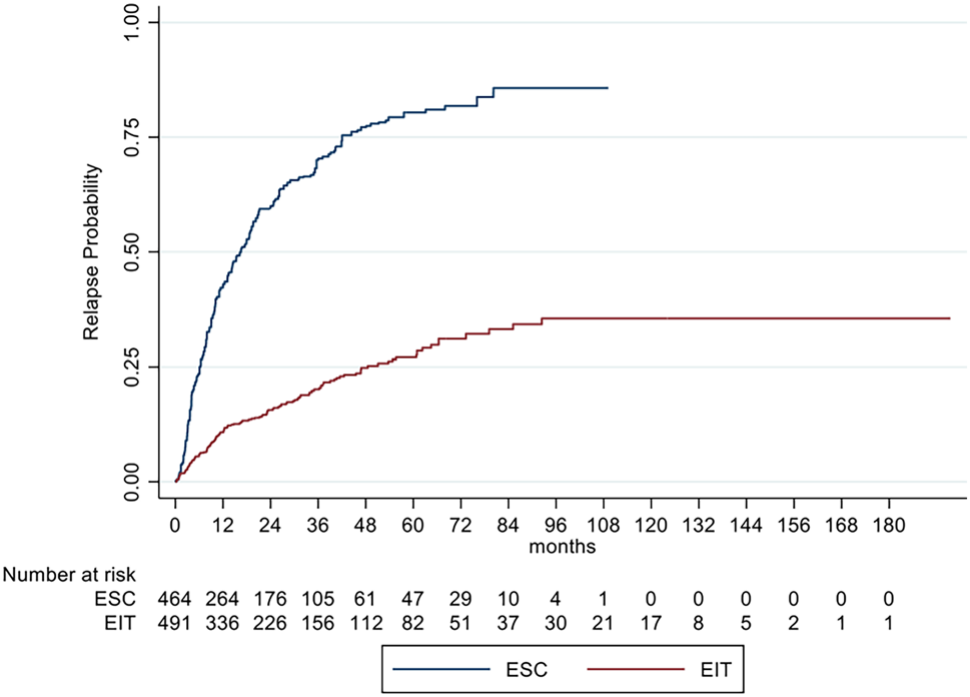
Cumulative probability for experiencing a relapse in RRMS patients comparing EIT and ESC patients

Mean annualized relapse rates of the unbalanced cohorts (ARR) were 0.12 (SD 0.42) for EIT and 0.42 (SD 0.49) for ESC and in the GLM model for relapses 0.09 (SE 0.01) and 0.4 (SE 0.03), respectively. The incidence rate ratio (IRR) in the GLM model for relapses showed a decreased relapse probability of 78% for EIT versus ESC patients (IRR = 0.22; 95% CI 0.16–0.30; *p* < 0.001). Analyzing the time to the first relapse by Cox regression, a hazard ratio of 0.17 (95% CI 0.13–0.22; *p* < 0.001) indicated a decreased risk for relapses of 83% for EIT patients (Fig. 1*, *Table 3). The mean annualized relapse rates with the GLM model revealed an ARR of 0.39 (SE 0.12) on treatment with DMF or TERI and an ARR of 0.04 (SE 0.02) after escalation of therapy.

**Table 3:** Main results in RRMS patients comparing EIT and ESC patients

	HR	95% Confidence interval—lower	95% Confidence interval—upper	Statistical significance (*p* value)
Relapse	0.17	0.13	0.22	** < 0.001**
Progression sustained for 12 weeks	0.55	0.40	0.76	** < 0.001**
Progression sustained for 24 weeks	0.55	0.39	0.78	** < 0.001**
Regression sustained for 12 weeks	1.03	0.71	1.50	0.878
Regression sustained for 24 weeks	1.08	0.71	1.64	0.717

Bold values represent *p* < 0.05

*EIT* early intensive treatment, *ESC* escalation, *HR* hazard ratio, *RRMS* relapsing–remitting multiple sclerosis

**Fig. 2:**
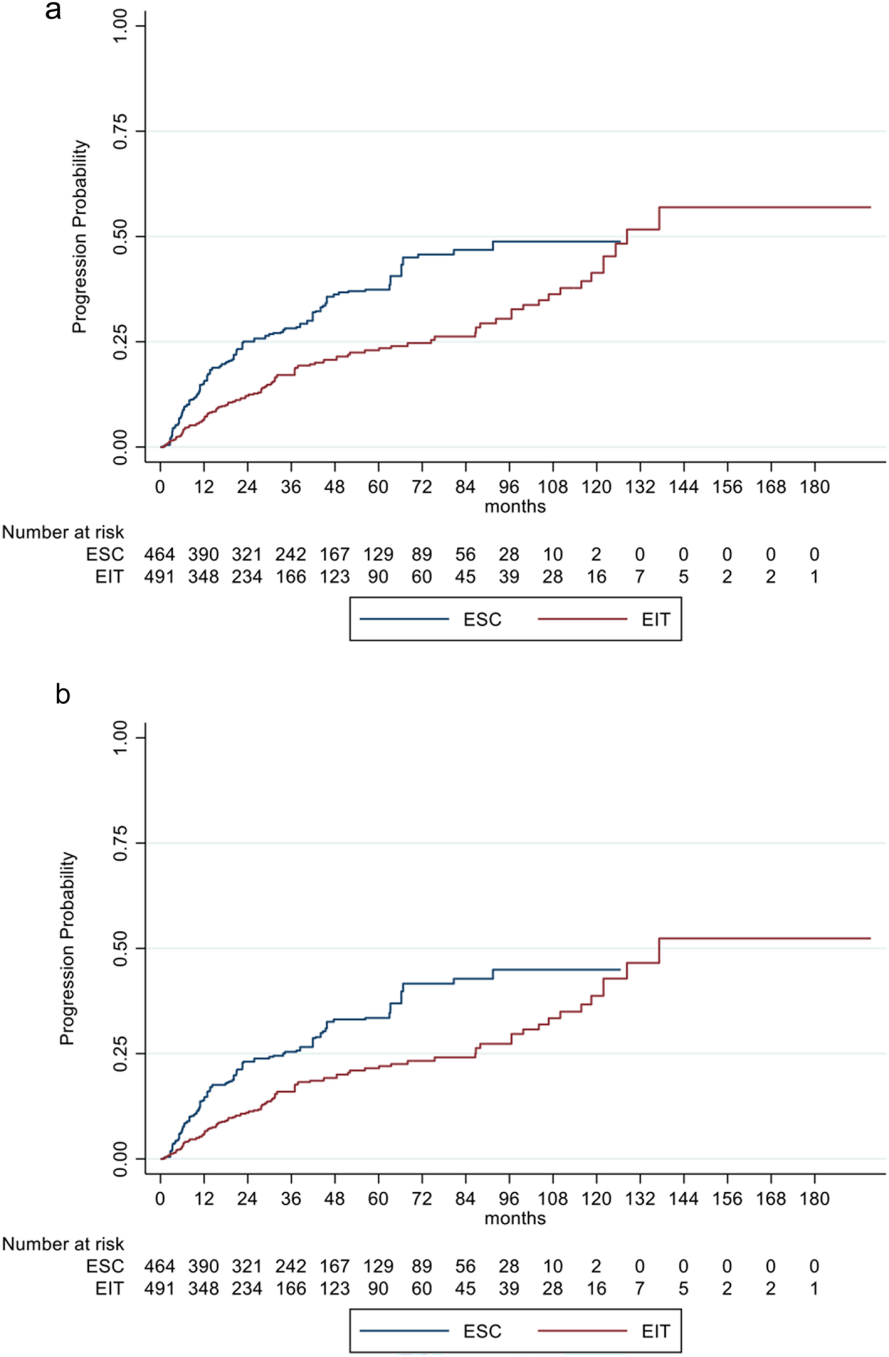
**a**, **b** Cumulative probability for disability progression sustained for 12 (**a**) and 24 weeks (**b**) in RRMS patients comparing EIT and ESC patients

EDSS progression, sustained for 12 weeks and 24 weeks, was significantly different between the two groups with a decreased risk of 45% (HR: 0.55, 95% CI 0.40–0.76; *p* < 0.001) and 45% (HR: 0.55, 95% CI 0.39–0.78; *p* < 0.001) for EIT patients, respectively (Fig. 2a, b*, *Table 3).

**Fig. 3:**
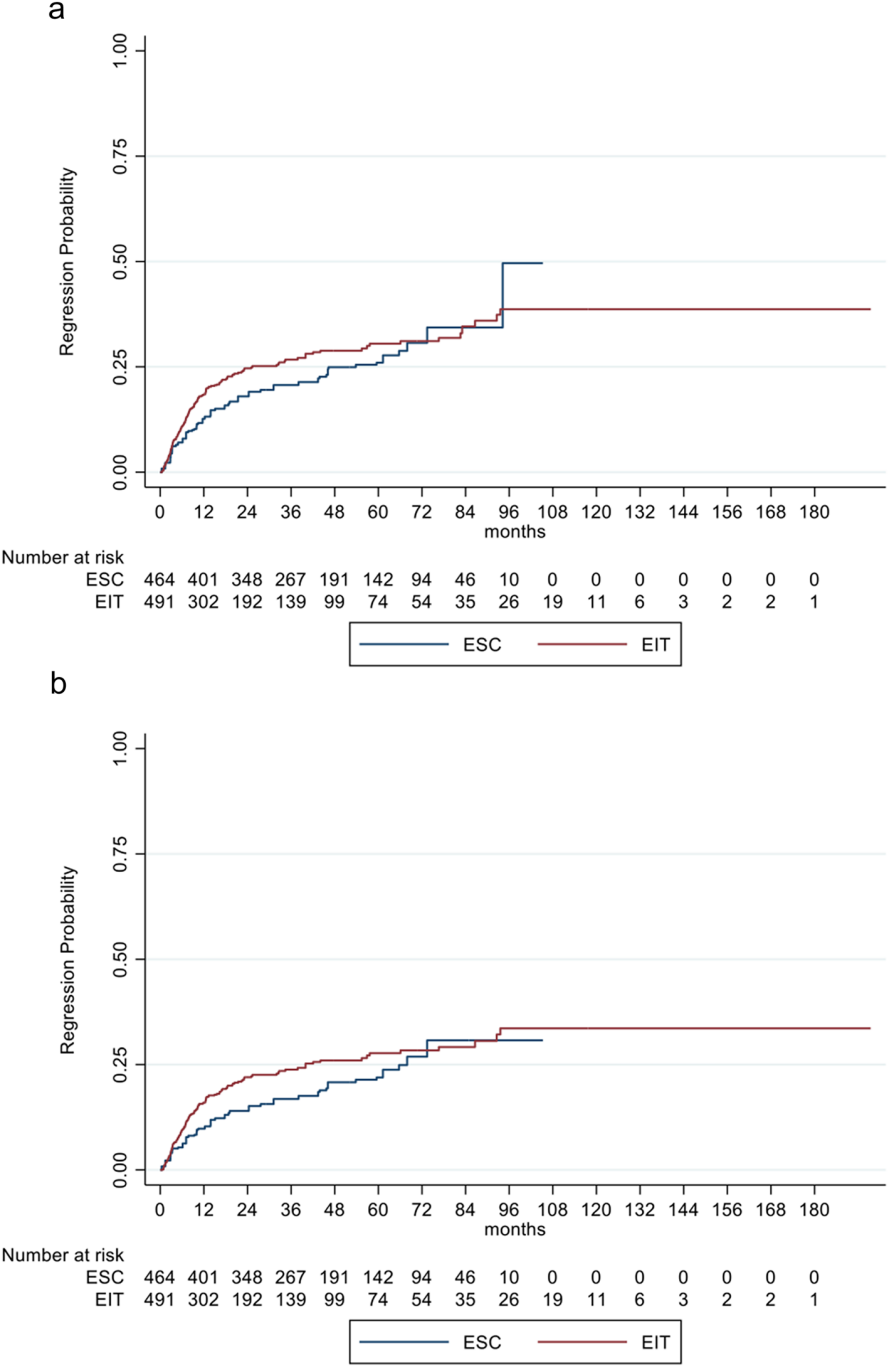
**a**, **b** Cumulative probability for disability regression sustained for 12 (**a**) and 24 weeks (**b**) in RRMS patients comparing EIT and ESC patients

Sustained EDSS regression for 12 and 24 weeks showed no significant difference in EIT versus ESC probands. For EIT patients, regression probability was non-significantly increased by 3% (HR: 1.03, 95% CI 0.71–1.50; *p* = 0.878) and 8% (HR: 1.08, 95% CI 0.72–1.64; *p* = 0.717) respectively, compared to ESC (Fig. 3a, b*, *Table 3).

The number of unbalanced patients interrupting treatment was 268 (36.1%) in EIT patients (147 stopped and 121 switched to another DMT) and 52 (23%) within ESC cohort (26 stopped and 26 switched to another DMT). The reasons for treatment interruption within the EIT group were mainly patients’ wishes (*n* = 95), disease progression (*n* = 86), stable disease course (defined at the physician’s and patient’s discretion; *n* = 36) and AEs (*n* = 34). Within the ESC cohort, the respective reasons were disease progression (*n* = 24), patients’ wishes (*n* = 18), stable disease course (*n* = 10), and AEs (*n* = 7). Pregnancy or the wish to conceive was documented in 31 patients in the EIT and 7 patients in the ESC cohort.

**Fig. 4:**
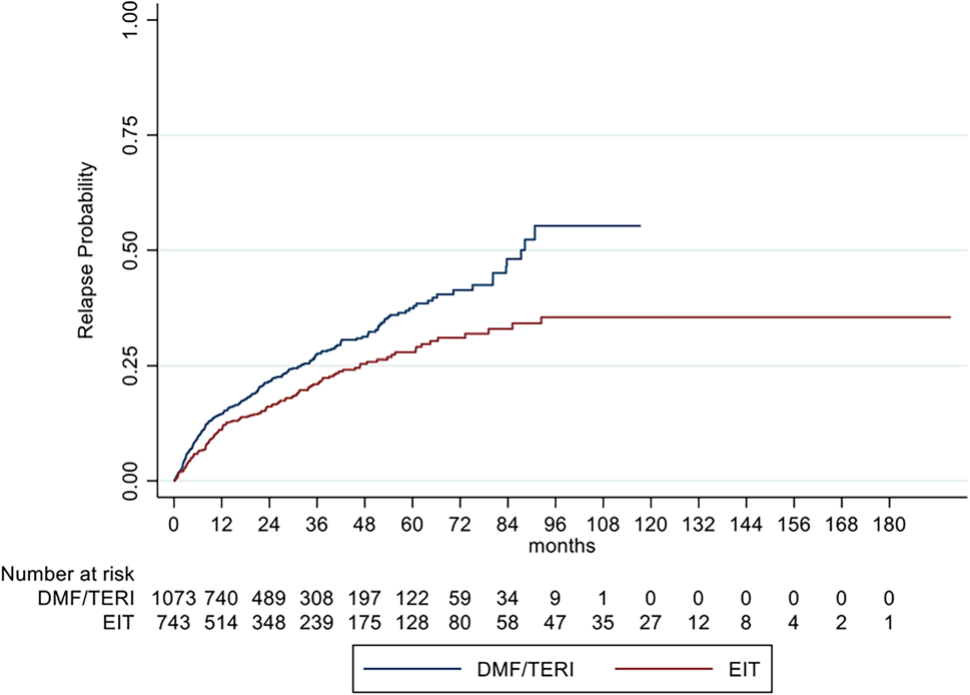
Cumulative probability for experiencing a relapse in RRMS patients comparing EIT and DMF/TERI patients

**Fig. 5:**
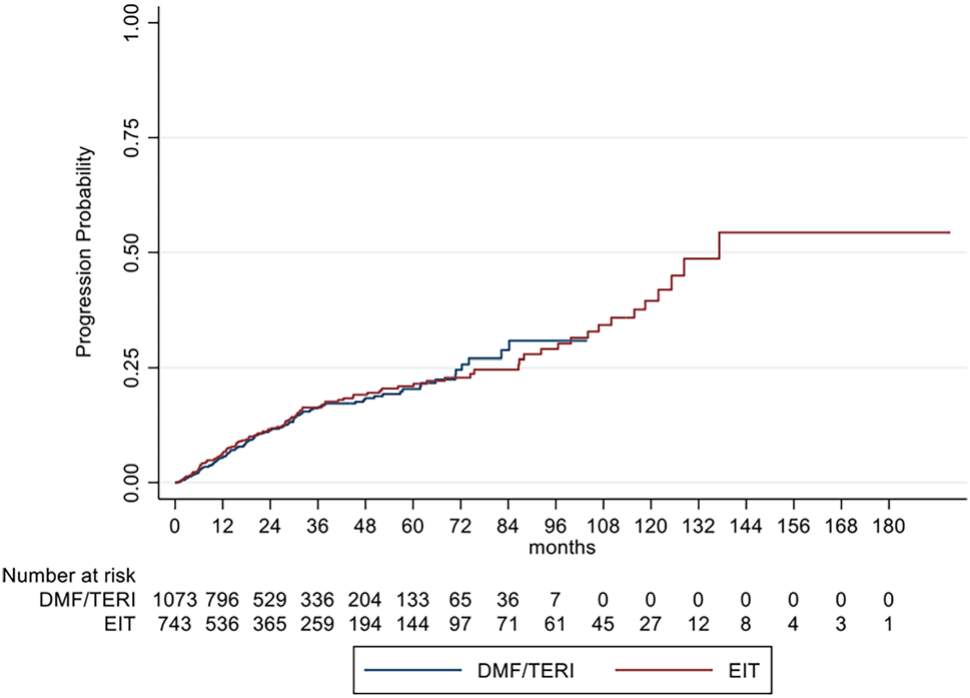
Cumulative probability for disability progression sustained for 12 weeks in RRMS patients comparing EIT and DMF/TERI patients

We also compared our EIT cohort with a group of RRMS patients with an initial treatment of DMF or TERI (DMF/TERI group) for at least 3 months to overcome a possible bias of investigating a highly active MS sub-cohort. The DMF/TERI group comprised 1073 patients for a mean follow-up period of 2.7 years (SD: 2.0) and an unbalanced ARR of 0.20 (SD: 0.54). After IPW the IRR in the GLM model for relapses showed a decreased relapse probability of 39% for EIT versus DMF/TERI patients (IRR = 0.61; 95% CI 0.48–0.79; *p* < 0.001). Analyzing the time to the first relapse by Cox regression, a HR of 0.50 (95% CI 0.40–0.63; *p* < 0.001) indicated a decreased risk for relapses of 50% for EIT patients (Fig. 4*, *Table 4). In contrast to our core analyses EIT versus ESC, sustained EDSS progression and regression for 12 and 24 weeks showed no benefit for EIT versus DMF/TERI patients (Fig. 5, Table 4). These data support the assumption that EIT is more effective in active to highly active than in mild to moderate active RRMS patients.

**Table 4:** Main results in RRMS patients comparing EIT and DMF/TERI patients

	HR	95% Confidence interval—lower	95% Confidence interval—upper	Statistical significance (*p* value)
Relapse probability	0.50	0.40	0.63	** < 0.001**
Progression sustained for 12 weeks	1.27	0.96	1.69	0.089
Progression sustained for 24 weeks	1.36	1.01	1.84	**0.042**
Regression sustained for 12 weeks	0.91	0.72	1.14	0.412
Regression sustained for 24 weeks	0.88	0.69	1.13	0.320

Bold values represent *p* < 0.05

*DMF* dimethylfumarate, *EIT* early intensive treatment, *ESC* escalation, *HR* hazard ratio, *RRMS* relapsing–remitting multiple sclerosis, *TERI* teriflunomide

## Discussion

In this observational study across a nation with well-defined MS treatment centers, we analyzed prospectively collected real-world data to compare the effectiveness of two different treatment algorithms, namely early intensive treatment (EIT) with AZM, CLAD, FTY, NTZ, OCR, or OZA and the escalation treatment (ESC) approach with AZM, CLAD, FTY, NTZ, OCR, or OZA after DMF or TERI therapy in large cohorts of RRMS patients. By nature, the different approved treatment labels caused slight differences in the baseline characteristics of the cohorts. To control for these, we used normalized IPW and provided well-balanced baseline parameters.

Previous studies revealed superior effectiveness comparing EIT versus ESC [17, 20, 27, 29–31, 34]. Harding et al. analyzed 592 RRMS patients in a cohort study with the primary outcome 5-year change in EDSS between EIT and ESC. This primary outcome was lower (i.e., more favorable) in the EIT group than the ESC group (0.3 vs 1.2). EIT was defined as treatment start with either AZM or NTZ, whereas FTY was classified as moderate efficacy treatment [17], which is in contrast to our study with FTY as highly effective treatment.

Spelman et al. retrieved data on 4861 patients from the Danish and Swedish national multiple sclerosis registries in a cohort study. The primary study outcome was time to 24-week confirmed disability worsening. The main difference between the two registries was, that in the Danish patients, in a total of 1994 (92.3%) a low to moderately effective DMT, mainly TERI (42.0%), had been initiated. In contrast, in the Swedish patients 1769 (65.5%) initiated a low to moderately effective DMT (TERI, 2.4%). In 931 (34.5%) a highly effective DMT, mainly rituximab (17.9%) had been initiated compared to 7.6% in the Danish cohort. The Swedish treatment strategy was associated with a 29% reduction in the rate of 24-week confirmed disability worsening relative to the Danish treatment strategy (*p* = 0.004) [34]. Iaffaldano et al. included 2702 RRMS patients from the Italian MS Registry in their cohort study comparing the EIT and ESC approach using disability trajectories for a median of 8.5 years. The EIT group comprised patients, who had received as first DMT FTY, NTZ, mitoxantrone, AZM, OCR or CLAD. Mean annual delta-EDSS changes were all significantly (*p* < 0.02) higher in the ESC group compared with the EIT group [20].

In an Argentinian cohort, EIT significantly decreased the risk of EDSS progression (*p* = 0.04), relapses (*p* = 0.006) and new MRI activity during follow-up (*p* < 0.001) [30]. Whereas, no significant differences were observed in specific adverse events between groups. EIT was defined as initial treatment with NTZ, OCR, rituximab, AZM, mitoxantrone, or CLAD.

Putting these findings into the perspective of our results, we also identified a lower probability of sustained EDSS progression and relapses for EIT patients compared to ESC. Differences to the aforementioned studies were that we also included OZA as novel treatment in the EIT approach but excluded mitoxantrone, cyclophosphamide and rituximab as highly effective treatments, as these are rarely or not anymore used in Austria. In addition, we defined an ESC regime as treatment change after DMF or TERI and did not consider interferon beta and glatiramer acetate as in aforementioned publications [17, 20, 29, 30], as these therapies are not documented in the AMSTR. Furthermore, the mean duration of 2.1 years until escalation after DMF and TERI was shorter than in most of the previous studies ranging from 2.4 to 6.3 years [17, 20, 29].

The ideal timepoint to start or switch DMT treatment is often difficult to decide or even predict. Ziemssen et al. proposed two windows of opportunity for treatment optimization in MS [36]. He postulated that both early initiation of treatment and prompt intervention if disease activity persists despite initial treatment are critical to optimize treatment outcomes. In line with these notions, several studies provided evidence for a better outcome in patients who had been escalated earlier [2, 16, 19, 26, 32]. In this context, our ESC approach with a shorter mean time period of 2.1 years to escalation is considered less disadvantageous regarding long-term disease progression in comparison with EIT algorithm. However, disease progression deteriorated significantly in the ESC group as compared with the EIT patients over 4.6 years in our study.

In summary, there is growing support for using EIT as the initial therapeutic approach in RRMS. However, much of this support is limited to observational real-world studies, while randomized control trials, such as DELIVER-MS, TREAT-MS, promise to provide systemically and prospectively collected evidence and more definitive answers [1, 23, 25, 33]. Hence, more real-world data analyses are needed.

The major strength of our study is the nationwide observational collection of relatively big and comprehensive data from approved MS clinics, comprising patients in Austria who have been treated with AZM, CLAD, FTY, NTZ, OCR or OZA first-line or after a treatment with DMF or TERI. The AMSTR represents a secure web-based platform that enables treating neurologists in all Austrian MS centers to immediately perform online documentation during patient visits. To ensure high documentation and data quality in terms of completeness and plausibility, the AMSTR is monitored by an external and independent clinical research organization, which represents another strength.

Some limitations have to be considered as well when interpreting our results. Information on MRI findings was only available at baseline before start of treatment, but we included this as an independent variable for propensity scoring and in the respective outcome models. We cannot provide information on imaging findings during follow-up. Furthermore, reasons for decision for the EIT or ESC approach could be systematically different in a way not captured by the propensity score, but clearly these cannot be captured by a data-base as used in the AMSTR. The treatment strategy in Austria is guided by expert statements for each therapy listed at the website of the AMSTR. These expert statements are in line with European treatment recommendations for MS [22, 35] and do also contain the specific reimbursement regulations in Austria.

Our study provides the first large analysis comparing an EIT strategy with AZM, CLAD, FTY, NTZ, OCR, or OZA and an ESC approach with AZM, CLAD, FTY, NTZ, OCR, or OZA after a treatment with DMF or TERI in RRMS patients. An escalation regime resulted in a higher relapse and sustained EDSS probability. Therefore, starting with a highly effective treatment should be preferred in patients with an active to highly active disease course. Given the currently available armamentarium of treatments and considering the findings of our study, an ESC approach currently shall only be considered in case of mild to moderate risk of disease activity.

## Data Availability

Data are available by request to the author.

## References

[1] Bou Rjeily N, Mowry EM, Ontaneda D, Carlson AK (2024). Highly effective therapy versus escalation approaches in early multiple sclerosis: what is the future of multiple sclerosis treatment?. Neurol Clin.

[2] Brown JWL, Coles A, Horakova D, Havrdova E, Izquierdo G, Prat A, Girard M, Duquette P, Trojano M, Lugaresi A, Bergamaschi R, Grammond P, Alroughani R, Hupperts R, McCombe P, Van Pesch V, Sola P, Ferraro D, Grand’Maison F (2019). Association of initial disease-modifying therapy with later conversion to secondary progressive multiple sclerosis. JAMA.

[3] Calabresi PA, Radue E-W, Goodin D, Jeffery D, Rammohan KW, Reder AT, Vollmer T, Agius MA, Kappos L, Stites T, Li B, Cappiello L, von Rosenstiel P, Lublin FD (2014). Safety and efficacy of fingolimod in patients with relapsing-remitting multiple sclerosis (FREEDOMS II): a double-blind, randomised, placebo-controlled, phase 3 trial. Lancet Neurol.

[4] Cohen JA, Coles AJ, Arnold DL, Confavreux C, Fox EJ, Hartung H-P, Havrdova E, Selmaj KW, Weiner HL, Fisher E, Brinar VV, Giovannoni G, Stojanovic M, Ertik BI, Lake SL, Margolin DH, Panzara MA, Compston DAS, CARE-MS I investigators (2012). Alemtuzumab versus interferon beta 1a as first-line treatment for patients with relapsing-remitting multiple sclerosis: a randomised controlled phase 3 trial. Lancet (London, England).

[5] Cohen JA, Comi G, Selmaj KW, Bar-Or A, Arnold DL, Steinman L, Hartung H-P, Montalban X, Kubala Havrdová E, Cree BAC, Sheffield JK, Minton N, Raghupathi K, Huang V, Kappos L, Trial RADIANCE, Investigators.  (2019). Safety and efficacy of ozanimod versus interferon beta-1a in relapsing multiple sclerosis (RADIANCE): a multicentre, randomised, 24-month, phase 3 trial. Lancet Neurol.

[6] Coles AJ, Twyman CL, Arnold DL, Cohen JA, Confavreux C, Fox EJ, Hartung H-P, Havrdova E, Selmaj KW, Weiner HL, Miller T, Fisher E, Sandbrink R, Lake SL, Margolin DH, Oyuela P, Panzara MA, Compston DAS, CARE-MS II investigators (2012). Alemtuzumab for patients with relapsing multiple sclerosis after disease-modifying therapy: a randomised controlled phase 3 trial. Lancet (London, England).

[7] Comi G, Kappos L, Selmaj KW, Bar-Or A, Arnold DL, Steinman L, Hartung H-P, Montalban X, Kubala Havrdová E, Cree BAC, Sheffield JK, Minton N, Raghupathi K, Ding N, Cohen JA, SUNBEAM Study Investigators (2019). Safety and efficacy of ozanimod versus interferon beta-1a in relapsing multiple sclerosis (SUNBEAM): a multicentre, randomised, minimum 12-month, phase 3 trial. Lancet Neurol.

[8] Confavreux C, O’Connor P, Comi G, Freedman MS, Miller AE, Olsson TP, Wolinsky JS, Bagulho T, Delhay J-L, Dukovic D, Truffinet P, Kappos L, TOWER Trial Group (2014). Oral teriflunomide for patients with relapsing multiple sclerosis (TOWER): a randomised, double-blind, placebo-controlled, phase 3 trial. Lancet Neurol.

[9] Fox RJ, Miller DH, Phillips JT, Hutchinson M, Havrdova E, Kita M, Yang M, Raghupathi K, Novas M, Sweetser MT, Viglietta V, Dawson KT (2012). Placebo-controlled phase 3 study of oral BG-12 or glatiramer in multiple sclerosis. N Engl J Med.

[10] Giovannoni G, Comi G, Cook S, Rammohan K, Rieckmann P, Soelberg Sørensen P, Vermersch P, Chang P, Hamlett A, Musch B, Greenberg SJ, CLARITY Study Group (2010). A placebo-controlled trial of oral cladribine for relapsing multiple sclerosis. N Engl J Med.

[11] Giovannoni G, Soelberg Sorensen P, Cook S, Rammohan K, Rieckmann P, Comi G, Dangond F, Adeniji AK, Vermersch P (2018). Safety and efficacy of cladribine tablets in patients with relapsing-remitting multiple sclerosis: results from the randomized extension trial of the CLARITY study. Mult Scler (Houndmills, Basingstoke, England).

[12] Glynn AN, Quinn K (2010). An introduction to the augmented inverse propensity weighted estimator. Polit Anal.

[13] Gold R, Kappos L, Arnold DL, Bar-Or A, Giovannoni G, Selmaj K, Tornatore C, Sweetser MT, Yang M, Sheikh SI, Dawson KT (2012). Placebo-controlled phase 3 study of oral BG-12 for relapsing multiple sclerosis. N Engl J Med.

[14] Guger M, Enzinger C, Leutmezer F, Kraus J, Kalcher S, Kvas E, Berger T (2018). Real-life clinical use of natalizumab and fingolimod in Austria. Acta Neurol Scand.

[15] Guger M, Enzinger C, Leutmezer F, Kraus J, Kalcher S, Kvas E, Berger T, Austrian MS Treatment Registry (AMSTR) (2020). Oral therapies for treatment of relapsing-remitting multiple sclerosis in Austria: a 2-year comparison using an inverse probability weighting method. J Neurol.

[16] Guger M, Enzinger C, Leutmezer F, Di Pauli F, Kraus J, Kalcher S, Kvas E, Berger T, Austrian MS Treatment Registry (AMSTR) (2023). Effects of horizontal versus vertical switching of disease-modifying treatment after platform drugs on disease activity in patients with relapsing-remitting multiple sclerosis in Austria. J Neurol.

[17] Harding K, Williams O, Willis M, Hrastelj J, Rimmer A, Joseph F, Tomassini V, Wardle M, Pickersgill T, Robertson N, Tallantyre E (2019). Clinical outcomes of escalation vs early intensive disease-modifying therapy in patients with multiple sclerosis. JAMA Neurol.

[18] Hauser SL, Bar-Or A, Comi G, Giovannoni G, Hartung H-P, Hemmer B, Lublin F, Montalban X, Rammohan KW, Selmaj K, Traboulsee A, Wolinsky JS, Arnold DL, Klingelschmitt G, Masterman D, Fontoura P, Belachew S, Chin P, Mairon N (2017). Ocrelizumab versus interferon beta-1a in relapsing multiple sclerosis. N Engl J Med.

[19] He A, Merkel B, Brown JWL, Zhovits Ryerson L, Kister I, Malpas CB, Sharmin S, Horakova D, Kubala Havrdova E, Spelman T, Izquierdo G, Eichau S, Trojano M, Lugaresi A, Hupperts R, Sola P, Ferraro D, Lycke J, Grand’Maison F (2020). Timing of high-efficacy therapy for multiple sclerosis: a retrospective observational cohort study. Lancet Neurol.

[20] Iaffaldano P, Lucisano G, Caputo F, Paolicelli D, Patti F, Zaffaroni M, Brescia Morra V, Pozzilli C, De Luca G, Inglese M, Salemi G, Maniscalco GT, Cocco E, Sola P, Lus G, Conte A, Amato MP, Granella F, Gasperini C (2021). Long-term disability trajectories in relapsing multiple sclerosis patients treated with early intensive or escalation treatment strategies. Ther Adv Neurol Disord.

[21] Kappos L, Radue E-W, O’Connor P, Polman C, Hohlfeld R, Calabresi P, Selmaj K, Agoropoulou C, Leyk M, Zhang-Auberson L, Burtin P, FREEDOMS Study Group (2010). A placebo-controlled trial of oral fingolimod in relapsing multiple sclerosis. N Engl J Med.

[22] Montalban X, Gold R, Thompson AJ, Otero-Romero S, Amato MP, Chandraratna D, Clanet M, Comi G, Derfuss T, Fazekas F, Hartung HP, Havrdova E, Hemmer B, Kappos L, Liblau R, Lubetzki C, Marcus E, Miller DH, Olsson T (2018). ECTRIMS/EAN guideline on the pharmacological treatment of people with multiple sclerosis. Eur J Neurol.

[23] Morgan A, Tallantyre E, Ontaneda D (2023). The benefits and risks of escalation versus early highly effective treatment in patients with multiple sclerosis. Expert Rev Neurother.

[24] O’Connor P, Wolinsky JS, Confavreux C, Comi G, Kappos L, Olsson TP, Benzerdjeb H, Truffinet P, Wang L, Miller A, Freedman MS, TEMSO Trial Group (2011). Randomized trial of oral teriflunomide for relapsing multiple sclerosis. N Engl J Med.

[25] Ontaneda D, Tallantyre EC, Raza PC, Planchon SM, Nakamura K, Miller D, Hersh C, Craner M, Bale C, Chaudhry B, Gunzler DD, Love TE, Gerry S, Coles A, Cohen JA, Evangelou N (2020). Determining the effectiveness of early intensive versus escalation approaches for the treatment of relapsing-remitting multiple sclerosis: the DELIVER-MS study protocol. Contemp Clin Trials.

[26] Ontaneda D, Mowry EM, Newsome SD, Naismith RT, Nicholas J, Fisher E, de Moor C, Bohn J, Ho P-R, Sandrock A, Rudick R, Williams JR (2022). Benefits of early treatment with natalizumab: a real-world study. Mult Scler Relat Disord.

[27] Pipek LZ, Mahler JV, Nascimento RFV, Apóstolos-Pereira SL, Silva GD, Callegaro D (2023). Cost, efficacy, and safety comparison between early intensive and escalating strategies for multiple sclerosis: a systematic review and meta-analysis. Mult Scler Relat Disord.

[28] Polman CH, O’Connor PW, Havrdova E, Hutchinson M, Kappos L, Miller DH, Phillips JT, Lublin FD, Giovannoni G, Wajgt A, Toal M, Lynn F, Panzara MA, Sandrock AW, Investigators AFFIRM (2006). A randomized, placebo-controlled trial of natalizumab for relapsing multiple sclerosis. N Engl J Med.

[29] Prosperini L, Mancinelli CR, Solaro CM, Nociti V, Haggiag S, Cordioli C, De Giglio L, De Rossi N, Galgani S, Rasia S, Ruggieri S, Tortorella C, Capra R, Mirabella M, Gasperini C (2020). Induction versus escalation in multiple sclerosis: a 10-year real world study. Neurotherapeutics.

[30] Rojas JI, Patrucco L, Alonso R, Garcea O, Deri N, Carnero Contentti E, Lopez PA, Pettinicchi JP, Caride A, Cristiano E (2022). Effectiveness and safety of early high-efficacy versus escalation therapy in relapsing-remitting multiple sclerosis in Argentina. Clin Neuropharmacol.

[31] Schmierer K, Sørensen PS, Baker D (2021). Highly effective disease-modifying treatment as initial MS therapy. Curr Opin Neurol.

[32] Simonsen CS, Flemmen HØ, Broch L, Brunborg C, Berg-Hansen P, Moen SM, Celius EG (2021). Early high efficacy treatment in multiple sclerosis is the best predictor of future disease activity over 1 and 2 years in a norwegian population-based registry. Front Neurol.

[33] Simpson A, Mowry EM, Newsome SD (2021). Early aggressive treatment approaches for multiple sclerosis. Curr Treat Opt Neurol.

[34] Spelman T, Magyari M, Piehl F, Svenningsson A, Rasmussen PV, Kant M, Sellebjerg F, Joensen H, Hillert J, Lycke J (2021). Treatment escalation vs immediate initiation of highly effective treatment for patients with relapsing-remitting multiple sclerosis: data from 2 different national strategies. JAMA Neurol.

[35] Wiendl H, Gold R, Berger T, Derfuss T, Linker R, Mäurer M, Aktas O, Baum K, Berghoff M, Bittner S, Chan A, Czaplinski A, Deisenhammer F, Di Pauli F, Du Pasquier R, Enzinger C, Fertl E, Gass A, Gehring K (2021). Multiple Sclerosis Therapy Consensus Group (MSTCG): position statement on disease-modifying therapies for multiple sclerosis (white paper). Ther Adv Neurol Disord.

[36] Ziemssen T, Derfuss T, de Stefano N, Giovannoni G, Palavra F, Tomic D, Vollmer T, Schippling S (2016). Optimizing treatment success in multiple sclerosis. J Neurol.

